# Limited predictive value of illness perceptions for short-term poor recovery in musculoskeletal pain. A multi-center longitudinal study

**DOI:** 10.1186/s12891-021-04366-7

**Published:** 2021-06-07

**Authors:** E. J. de Raaij, H. Wittink, J. F. Maissan, P. Westers, R. W. J. G. Ostelo

**Affiliations:** 1 Research Group Lifestyle and Health, HU University of Applied Sciences Utrecht, Heidelberglaan 7, CS Utrecht, 3584 The Netherlands; 2 Department of Health Sciences, VU University, Amsterdam, The Netherlands; 3 the EMGO Institute for Health and Care Research, Amsterdam, The Netherlands; 4 Julius Center for Health Sciences and Primary Care, Department of Data Science and Biostatistics, Utrecht University, Utrecht, Netherlands; 5 Department of Epidemiology and Biostatistics, VU University Medical Centre, Amsterdam, The Netherlands

**Keywords:** Illness perceptions, Musculoskeletal pain, Prediction poor outcome, Pain, Physical functioning

## Abstract

**Background:**

Musculoskeletal pain (MSP) is recognized worldwide as a major cause of increased years lived with disability. In addition to known generic prognostic factors, illness perceptions (IPs) may have predictive value for poor recovery in MSP. We were interested in the added predictive value of baseline IPs, over and above the known generic prognostic factors, on clinical recovery from MSP. Also, it is hypothesized there may be overlap between IPs and domains covered by the Four-Dimensional Symptom Questionnaire (4DSQ), measuring distress, depression, anxiety and somatization. The aim of this study is twofold; 1) to assess the added predictive value of IPs for poor recovery and 2) to assess differences in predictive value for poor recovery between the Brief Illness Perception Questionnaire - Dutch Language Version (Brief IPQ-DLV) and the 4DSQ.

**Methods:**

An eligible sample of 251 patients with musculoskeletal pain attending outpatient physical therapy were included in a multi-center longitudinal cohort study. Pain intensity, physical functioning and Global Perceived Effect were the primary outcomes. Hierarchical logistic regression models were used to assess the added value of baseline IPs for predicting poor recovery. To investigate the performance of the models, the levels of calibration (Hosmer-Lemeshov test) and discrimination (Area under the Curve (AUC)) were assessed.

**Results:**

Baseline ‘Treatment Control’ added little predictive value for poor recovery in pain intensity [Odds Ratio (OR) 0.80 (Confidence Interval (CI) 0.66–0.97), increase in AUC 2%] and global perceived effect [OR 0.78 (CI 0.65–0.93), increase in AUC 3%]. Baseline ‘Timeline’ added little predictive value for poor recovery in physical functioning [OR 1.16 (CI 1.03–1.30), increase in AUC 2%]. There was a non-significant difference between AUCs in predictive value for poor recovery between the Brief IPQ-DLV and the 4DSQ.

**Conclusions:**

Based on the findings of this explorative study, assessing baseline IPs, over and above the known generic prognostic factors, does not result in a substantial improvement in the prediction of poor recovery.

Also, no recommendations can be given for preferring either the 4DSQ or the Brief IPQ-DLV to assess psychological factors.

## Introduction

Musculoskeletal pain (MSP) is a major cause of increased years lived with disability [1]. There are several generic factors prognostic of poor recovery from MSP [2]: widespread pain (≥ 2 pain sites), high functional disability, somatization, and high pain intensity. Psychological factors such as distress, depressive mood and somatization have also been identified as risk factors for the transition from acute to chronic low back pain [3–6]. These domains have been identified, but no recommendation can be made as to the best instrument for identifying these factors. In The Netherlands, the Four-Dimensional Symptom Questionnaire (4DSQ) is commonly used to assess distress, depression, anxiety and somatization [7]. In addition, illness perceptions (IPs), as the core element of the Common-Sense Model of Self-regulation of Health and Illness (CSM), have been recognized as possible risk factors for poor recovery from MSP. The Brief Illness Perceptions Questionnaire (Brief IPQ) is frequently used to assess these IPs [8]. A recent systematic review showed limited to moderate evidence for the association of some IPs with pain intensity (PI) and physical functioning (PF) in MSP [9]. Pathways by which these associations can influence MSP are not known. IPs might act as moderators or mediators or affect MSP through fear avoidance or catastrophizing. Another important finding of the review was that longitudinal research is lacking. Therefore, it is desirable to explore the added predictive value of IPs, over and above the well-known generic factors for poor recovery from MSP, in the physiotherapy setting.

The CSM model provides a framework for identifying unhelpful cognitions and emotions people may have about their MSP condition [10]. It is based on a parallel processing model, describing individual representations (i.e. IPs) in response to health threats (i.e. MSP). There are 9 IP dimensions included in the CSM: Consequences, Timeline, Personal Control, Treatment Control, Identity, Concern, Coherence, Emotional Response, and Causal [11, 12].

To investigate the added predictive value of IPs, we used the term ‘predictor’ defined as: “A patient characteristic that identifies subgroups of treated patients having different outcomes” [13]. In our study, IPs were seen as predictors, the treatment was usual care physiotherapy, and the disease was non-specific MSP.

Previous research has found that IPs are predictive for and associated with psychological factors, such as depression and anxiety, in patients with fibromyalgia [14], chronic back pain [15] systemic lupus erythematosus [16] and informal carers of patients with depression [17]. Therefore, overlap may exist between the domains included in the 4DSQ and in the Brief IPQ. Because of this potential overlap, we were interested in the correlation of these questionnaires. We were also interested in the difference between the added predictive values of the 4DSQ and the Dutch language version of the Brief IPQ (Brief IPQ-DLV) for poor recovery.

The following are our three research goals; First, to what extent do baseline illness perceptions in MSP patients have added predictive value for poor recovery in PI, PF and patient GPE after 3 months? Second, what is the correlation between the 4DSQ and the BIPQ-DLV? Third, what is the difference in added predictive value for poor recovery between the 4DSQ and the BIPQ-DLV?

## Method

### Design and setting

Twenty-eight primary care physiotherapy centres participated in this five-month-long exploratory study, approved by the Medical Ethical Committee of the University of Applied Sciences Utrecht (HU) (Ref. no. 430012019). Physiotherapists at these centres collected the data as part of their HU Master of Physiotherapy study. All participating patients were treated according to the Good Clinical Practice guidelines [18].

A consecutive sample of patients attending outpatient physiotherapy was invited at first contact by participating physiotherapists to take part. As part of an assignment in their master’s program, these physiotherapists included in the study 10–30 consecutive patients over a period of 2 months (after screening for in- and exclusion criteria: Table 1). After baseline (T0) assessment, a follow-up assessment after 3 months (T1) was performed, using a questionnaire assessing the dependent and independent variables (see Measurements).

**Table 1:** Inclusion criteria

- Musculoskeletal pain	
Joint conditions (i.e. rheumatoid arthritis (RA), osteoarthritis (OA)), bone conditions (i.e. osteoporosis), spinal disorders (e.g. low back pain), regional and widespread pain disorders, musculoskeletal injuries, multisystem inflammatory diseases	
- Age 18–75 years	
- No physiotherapy treatment in the 6 months before baseline	
- Signed informed consent	
- No serious musculoskeletal diseases	
° Fractures, malignancy, neurological signs	

Patients who met the inclusion criteria and gave written informed consent were recruited. We defined MSP as: Pain felt within the context of the musculoskeletal conditions listed in Table 1, according to the European Musculoskeletal Conditions Surveillance and Information Network.

All clinical procedures used in this study were carried out in accordance with relevant guidelines and regulations of the Royal Dutch Society of Physiotherapy (KNGF).

### Measurements

At baseline (T0), we collected data on demographic characteristics and the independent variables listed below:

#### Independent variables

- Pain intensity (PI)
- Average pain in the last 24 h (11-point Numeric Rating Scale (NRS): 0 = no pain; 10 = worst pain imaginable) [19].
- Physical functioning (PF)
- Difficulty in performing daily activities (11-point Patient-Specific Functional Scale (PSFS): 0 = no difficulty; 10 = unable to perform the activity). The PSFS is reportedly feasible and reliable [20, 21].
- Pain duration
- Patients rated how long their pain had existed prior to consultation: 1: pain < 7 weeks; 2: pain 7–13 weeks; 3: > 13 weeks.
- Number of pain sites
- Based on patients’ reports, the number of different pain sites were categorized as: 1: 1–2 sites; 2: > 2 sites.
- Psychological measures
- The Four-Dimensional Symptom Questionnaire (4DSQ) was used to assess patients’ level of risk (low, medium or high) for developing Distress (16 items), Somatization (16 items), Anxiety (12 items), and Depression (6 items). The 4DSQ is suitable for clinical applications. The items are answered on a 5-point frequency scale. To calculate sum scores, responses are coded on a 3-point scale: “no” (0 points), “sometimes” (1 point), “regularly”, “often”, and “very often or constantly” (2 points). Then, sum scores are calculated for each dimension, and cut-off points applied to categorize each patient as at low, medium or high risk [7].
- Illness perceptions
- The cross-cultural adapted and validated Brief Illness Perceptions Questionnaire- Dutch language Version (IPQ-DLV) was used [22, 23]: this consists of nine questions of which eight were scored on an 11-point scale and cover the IP dimensions of Consequences, Timeline, Personal Control, Treatment Control, Identity, Concern, Coherence, and Emotional Response. The IP dimensions of control beliefs (Personal/Treatment) and Coherence were converted before statistical analyses as they are scored in reverse. Higher scores on Brief IPQ-DLV were theorized to have a greater chance on poor recovery. The ninth IP question, the Causal dimension, has rank-ordered free-text responses and was not added as a predictor.

#### Dependent variables

For Global Perceived Effect (GPE), we used a 7-point scale ranging from ‘completely recovered’ to ‘very much worsened’. The GPE is a reliable measurement [24] with a clinically meaningful improvement cut-off point at ≤2 on a 7-point scale [25].

We defined poor recovery in three different ways [26];

- PI at follow-up; score of ≥3 on an 11-point NRS (0–10)
- PF at follow-up; score of ≥3 on an 11-point NRS (0–10)
- GPE; score of ≥3 on a 7-point ordinal scale

Pain intensity and physical function were also assessed at T1 together with the Global Perceived Effect.

### Statistics

In addition to age and gender, baseline scores were assessed for PI, PSFS, pain duration, number of pain sites, the 4DSQ, and the Brief IPQ-DLV, as percentages or means (standard deviation (SD)).

Hierarchical logistic regression models were constructed to examine the added predictive value of baseline ‘poor recovery’ (at 3 months). In the first block, age, gender and baseline scores for generic prognostic factors (psychological measures, PI, limitations in PF, number of pain sites and duration of pain) were entered as fixed (independent) variables. In the second block, baseline IPs with univariate significant ORs (*p* <  0.10) were added to the model. The final model was obtained by using the backward stepwise method. The goodness-of-fit of the model was described by the Nagelkerke *R*^2^ and the Receiver Operating Characteristics (ROC) curve with Area Under the Curve (AUC). Goodness-of-fit of the AUC was judged thus: 0.90–1.0 Excellent; 0.80–0.89 Good; 0.70–0.79 Fair; 0.60–0.69 Poor; 0.50–0.59 Fail. For calibration, we checked the goodness-of-fit using the Hosmer & Lemeshow test (*p* <  0.05). The SPSS package 25™ was used to analyze the data.

For our research question ‘*Is there an association between the 4DSQ and the BIPQ-DLV?*’, we used the non-parametric Spearman’s rank correlation coefficient. To interpret the strength of the correlation, we used the following classification; 0.00–0.10 negligible, 0.10–0**.39** weak, 0.40–0.69 moderate, 0.70–0.89 strong and 0.90–1.00 very strong [27].

For our research question ‘*Is there a difference in added predictive value of poor recovery between the 4DSQ and the BIPQ-DLV*?’, two regression models were built to examine the predictive value of baseline ‘poor recovery’ (at 3 months). In our first model, we entered age, gender and the baseline scores for generic prognostic factors (PI, limitations in PF, number of pain sites and duration of pain) and added the baseline score of the 4DSQ. In our second model, we replaced the 4DSQ with the Brief-IPQ-DLV. To test the discrimination of the each model, a ROC-curve with Area Under the Curve (AUC) was applied. To compare the two AUCs, we used the empirical (non-parametric) method with NCSS 2020 software.

## Results

A total of 251 (N_max_) participants was included in this study (see Table 2). We found missing data to be Missing Completely at Random (Little’s MCAR test *p* > 0*.*05). Numbers of missing items are reported in Table 3 in the ‘n’ column. A total of 237 participants was present at follow-up. The baseline characteristics of the fourteen participants lost to follow-up are described in Table 2 last column.

**Table 2:** Demographic characteristics, baseline generic prognostic baseline factors and baseline illness perceptions *N* = 251

		Lost to follow-up *N* = 14
Age (SD)	46.1 (13.8)	41.3 (13.7)
Gender ♀ (%)	68.9	85.7
Body pain locations (%)		
Head	4.7	0.0
Neck, shoulder, upper spins	35.6	50.0
Elbow, pols, hand	3.8	7.1
Lower back	16.5	21.4
Hip, knee	14.8	0.0
Ankle, foot	5.5	7.2
Multiple locations	19.1	14.3
Musculoskeletal pain conditions (%) ***N*** = 192		
Joint conditions (i.e. rheumatoid arthritis	2.1	0
Osteoarthritis	18.2	25.0
Bone conditions (i.e. osteoporosis)	3.1	0
Musculoskeletal injuries (e.g. low back pain)	64.1	70.0
Regional and widespread pain disorders	12.5	0
Multisystem inflammatory diseases	0	0
Pain intensity 0–10 (SD)	6.3 (2.8)	7.0 (2.4)
Physical functioning (0–10)	6.3 (2.2)	6.2 (1.5)
Pain duration %		
< 7 weeks	32.3	28.6
7–13 weeks	20.7	7.1
> 13 weeks	47.0	64.3
> 2 pain sites (%)	19.1	14.3
4DSQ risk of (%)		
Somatization (%)		
Low (0–10)	59.8	50.0
Medium [11–20]	29.5	41.7
High [21–32]	10.7	8.3
Distress (%)		
Low (0–10)	61.2	72.7
Medium [11–20]	22.7	9.1
High [21–32]	16.1	18.2
Anxiety (%)		
Low (0–3)	75.4	69.2
Medium [4–9]	10.3	23.1
High [10–24]	14.3	7.7
Depression (%)		
Low (0–2)	81.5	61.5
Medium [3–5]	7.3	23.1
High [6–12]	11.3	15.4
Baseline illness perceptions 0–10 (SD)		
Consequences	5.4 (2.9)	5.1 (3.8)
Timeline	5.1 (3.2)	4.1 (3.0)
Personal Control^a^	4.8 (2.6)	4.4 (3.7)
Treatment Control^a^	7.3 (2.1)	6.1 (3.3)
Identity	5.8 (2.3)	5.9 (3.2)
Concern	4.1 (3.6)	5.1 (3.7)
Coherence^a^	6.8 (2.5)	6.0 (3.6)
Emotional Response	4.5 (3.1)	4.9 (3.9)

*SD* standard deviation, *4DSQ* Four-Dimensional Symptom Questionnaire, ^a^ reversed score

**Table 3:** Missing values analyses

	N	Mean	SD	n	%
T0 Pain Intensity	245	6.3	2.3	6	2.4
T1 Pain Intensity	233	2.6	2.2	18	7.2
T0 Patient-Specific Functioning Scale	244	6.3	2.1	7	2.8
T1 Patient-Specific Functioning Scale	224	3.3	2.6	17	7.6
Global Perceived Effect	224			17	10.8

*N* = number of respondents, *SD* Standard Deviation, *n* number of non-respondents;

MCAR test *p* > 0.05

We found poor clinical recovery in 79 out of 204 participants (39%) for PI, 109 out of 200 (54.5%) for PF, and 59 out of 199(30%) for GPE.

For distribution of the generic prognostic factors according with baseline IPs for good or poor recovery, see Table 4.

**Table 4:** Distribution of generic prognostic factors at baseline according to good/poor clinical recovery

	Pain intensity recovery		Physical Functioning recovery		Global Perceived Effect recovery	
	Good *N* = 140	Poor *N* = 93	Good *N* = 99	Poor *N* = 125	Good *N* = 54	Poor *N* = 180
Pain intensity 0–10 (SD)	6.3 (2.2)	6.3 (2.2)	6.4 (2.0)	6.2 (2.5)	6.4 (2.2)	6.1 (2.4)
Physical functioning (0–10)	6.0 (2.5)	6.1 (2.3)	6.6 (2.0)	6.0 (2.1)	6.4 (2.2)	6.1 (2.2)
Pain duration %						
< 7 weeks	38.7	14.6	40.8	16.9	41.8	18.3
7–13 weeks	21.0	25.0	17.7	31.0	18.3	29.6
> 13 weeks	40.3	60.4	41.5	52.1	39.9	52.1
> 2 pain sites (%)	14.9	35.4	14.3	28.2	15.0	29.6
4DSQ risk of (%)						
Somatization						
Low	64.8	40.4	65.7	51.4	64.0	53.7
Medium	25.0	46.8	24.5	34.3	27.3	31.3
High	10.2	12.2	9.8	14.3	8.7	14.9
Distress						
Low	62.1	53.2	63.6	54.9	62.4	58.6
Medium	22.6	27.7	23.1	25.4	26.2	20.0
High	15.3	19.1	13.3	19.7	11.4	21.4
Anxiety						
Low	76.6	70.8	79.0	68.6	78.5	71.4
Medium	8.0	16.7	7.7	14.3	10.1	7.1
High	15.4	12.5	13.3	17.1	11.4	21.4
Depression						
Low	82.7	81.3	84.2	80.3	85.5	77.5
Medium	6.7	6.3	6.2	7.0	6.6	7.0
High	10.6	12.5	9.6	12.7	7.9	15.5
IPs 0–10 (SD)						
Consequences	5.2 (2.8)	6.0 (2.9)	5.3 (2.8)	5.8 (2.9)	5.2 (2.9)	5.7 (2.6)
Timeline	4.9 (3.3)	6.2 (3.0)	4.7 (3.2)	6.3 (3.2)	4.7 (3.3)	6.4 (3.0)
Personal Control^a^	4.9 (2.6)	4.7 (2.2)	4.8 (2.4)	4.7 (2.6)	4.9 (2.6)	4.9 (2.5)
Treatment Control^a^	7.5 (1.9)	7.1 (2.4)	7.5 (1.9)	7.0 (3.0)	7.6 (1.9)	6.6 (2.1)
Identity	5.7 (2.3)	6.3 (2.3)	5.9 (2.2)	6.1 (2.3)	5.2 (2.3)	6.0 (2.3)
Concern	3.4 (3.0)	4.8 (3.0)	3.9 (2.9)	4.3 (3.1)	3.9 (3.0)	4.6 (3.0)
Coherence^a^	7.0 (2.5)	6.6 (2.1)	6.9 (2.6)	7.0 (1.9)	6.9 (2.6)	6.8 (2.1)
Emotional Response	4.1 (3.0)	5.6 (3.0)	4.0 (3.0)	5.2 (3.0)	4.1 (3.0)	4.9 (2.9)

*SD* standard deviation, *4DSQ* Four-Dimensional Symptom Questionnaire, *IP* illness perception. ^a^ Scoring reversed

### Univariate logistic regression of illness perceptions with poor clinical recovery

Table 5 shows the results of the univariate logistic regression of baseline IPs with poor clinical recovery.

**Table 5:** Univariate associations of baseline illness perceptions with poor recovery: *N* = 251

T0 IP dimension	PI *N* = 221			PF *N* = 212			GPE *N* = 222		
	OR	CI	*p*	OR	CI	*p*	OR	CI	*p*
Consequences	1.1	1.0–1.2	**.021**	1.1	1.0–1.2	**.016**	1.2	1.1–1.3	.**004**
Timeline	1.1	1.0–1.2	.**007**	1.2	1.1–1.3	**.000**	1.2	1.1–1.4	.**000**
Personal Control	.98	.88–1.1	.686	.98	.88–1.1	.746	1.0	.89–1.1	.896
Treatment Control	.82	.71–.94	.**004**	.96	.84–1.1	.581	.76	.63–.96	.**004**
Identity	1.2	1.0–1.3	**.009**	1.2	1.0–1.3	**.015**	1.2	1.0–1.3	.**042**
Concern	1.2	1.1–1.3	**.000**	1.1	1.0–1.2	**.011**	1.2	1.1–1.3	**.003**
Coherence	.85	.76–.95	**.005**	.93	.83–1.1	.196	.93	.82–1.1	.296
Emotional Response	1.2	1.1–1.3	**.000**	1.2	1.1–1.3	**.002**	1.1	1.0–1.3	**.018**

*IP* illness perception, *GPE* Global Perceived Effect, *CI* Confidence interval

*p* = 0.05, Bold = threshold *p* <  0.10

For the hierarchical model, the following IP dimensions were statistically significant and were therefore selected for entering in Block 2: for the clinical outcome PI, Timeline, Treatment Control, Identity, Concern, Coherence and Emotional Response; for PF, Consequences, Timeline, Identity, Concern and Emotional Response; for GPE, Consequences, Timeline, Treatment Control, Identity, Concern and Emotional Response.

### Hierarchical logistic regression for baseline illness perceptions predicting poor recovery at 3 months

Table 6 shows results of the hierarchical logistic regressions and the AUC. Entered as fixed variables in Block 1 for all regression models were age, gender and generic prognostic factors. In Block 2 of the model, we added all the univariate significantly associated IPs (see Table 5) with the backward stepwise method. We report only the final models.

**Table 6:**
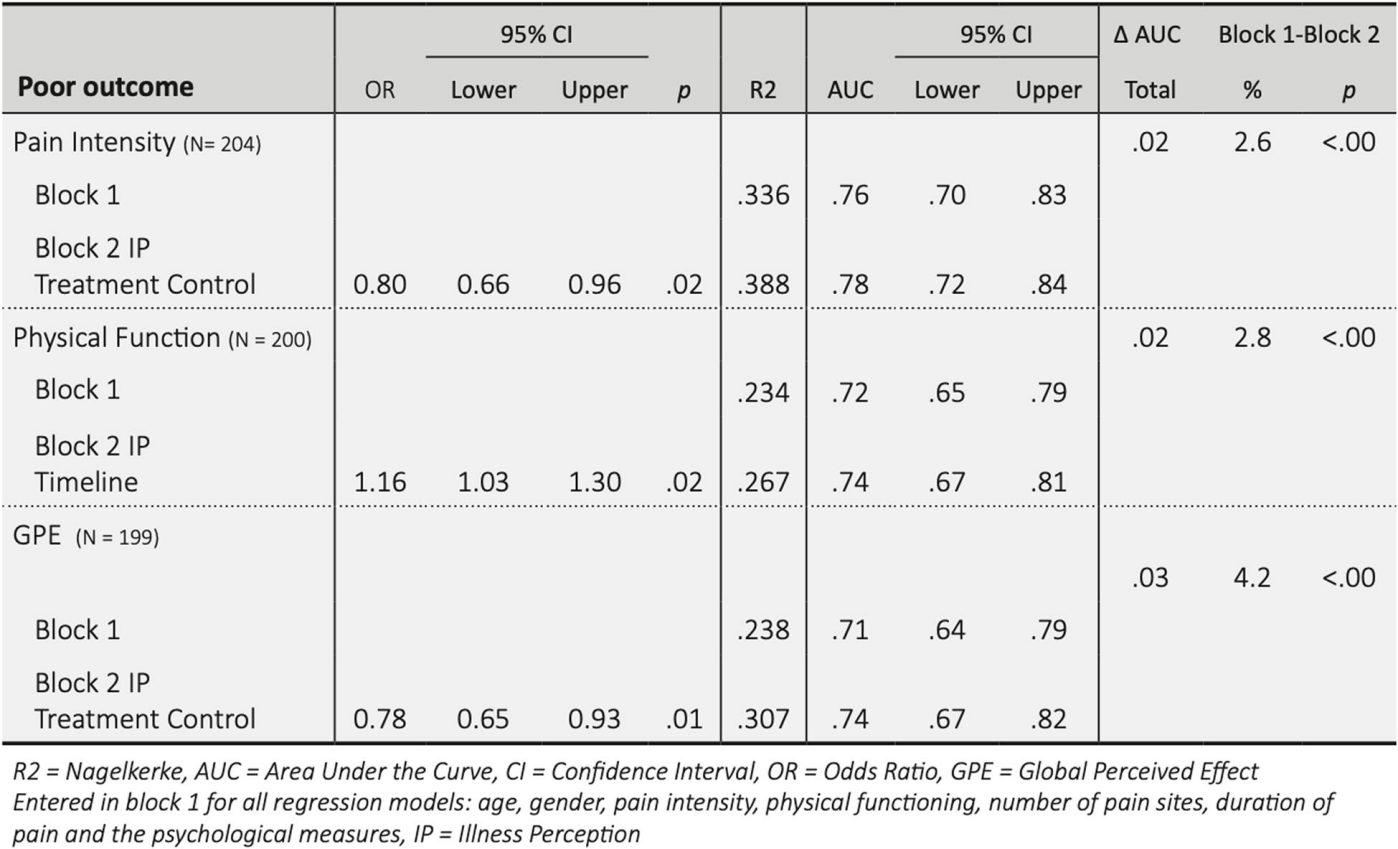
final hierarchical logistic regression models for predicting poor recovery at 3 months and added predictive probability value (AUC) IPs for poor outcome (N_max_ = 251)

#### Baseline IPs

After being added to Block 2, most IP dimensions did not increase predictive values for poor outcomes on PI, PF or GPE. Two IP dimensions did add predictive value: lower scores on Treatment Control for PI and GPE; and a higher score on Timeline for PF. The discrimination of each model after adding IPs increased slightly (the AUC increased by 2–3%). The goodness-of-fit was adequate (Hosmer & Lemeshow test (PI: *p* = 0.57; PSFS: *p* = 0.68; GPE: *p* = .08)).

#### Association of baseline scores in 4DSQ with the brief IPQ-DLV

The Spearman rank correlations showed small associations between the Brief IPQ-DLV and the 4DSQ. The IP dimensions ‘Personal Control’, ‘Treatment Control’ and ‘Coherence’ showed non-significant associations (Table 7).

**Table 7:** Baseline Spearman’s correlations for the Brief IPQ-DLV with the 4DSQ

IP dimension	Distress	Anxiety	Depression	Somatization
Consequences	0.37^a^	0.37^a^	0.34^a^	0.32^a^
Timeline	0.25^a^	0.20^a^	0.22^a^	0.32^a^
Personal Control	0.01	0.01	0.01	−0.01
Treatment Control	−0.01	0.02	0.00	0.03
Identity	0.26^a^	0.24^a^	0.25^a^	0.25^a^
Concern	0.27^a^	0.22^a^	0.27^a^	0.32^a^
Coherence	0.11	0.07	0.12	0.10
Emotional Response	0.40^a^	0.34^a^	0.34^a^	0.38^a^

^a^ Correlation significant at the ≤ 0.01 level (2-tailed)

### Difference in predictive value of poor recovery between the brief IPQ-DLV and the 4DSQ

Table 8 presents the predictive value of poor recovery between the Brief IPQ-DLV and the 4DSQ.

**Table 8:** Difference in predictive value of poor recovery between Brief IPQ-DLV and 4DSQ (N_max_ = 251)

	4DSQ 95% CI			Brief IPQ-DLV 95% CI			∆ AUC1-AUC2		
	AUC1	Lower	Upper	AUC2	Lower	Upper	Absolute	%	*p*
PI (*N* = 204)	0.65	0.54	0.74	0.63	0.50	0.73	0.03	4.0	0.61
PF (*N* = 200)	0.62	0.53	0.69	0.59	0.49	0.67	0.04	4.4	0.50
GPE (*N* = 199)	0.67	0.57	0.74	0.68	0.59	0.76	0.01	1.9	0.72

*AUC* Area Under the Curve, *CI* Confidence Interval, *PI* pain intensity, *PF* physical functioning, *GPE* Global Perceived Effect

## Discussion

In addition to generic prognostic factors, two of the IP dimensions, Treatment Control and Timeline, give a small added predictive value for poor recovery from MSP in pain intensity, physical functioning and Global Perceived Effect. The Brief IPQ-DLV showed weak correlation with the 4DSQ for all IP dimensions. The highest correlations (0.32 to 0.40) were for the IP dimensions Consequences and Emotional Response. There were no significant differences in the added predictive values for poor recovery between the Brief IPQ-DLV and the 4DSQ.

### Added predictive value of illness perceptions

Most IPs did not add predictive value for poor recovery. The amount of explained variance in Block 1 increased when adding Block 2 (Table 6) but the increase was small and most of the variance remained unexplained. This is also seen in the increase of the AUC from Step 1 to 2 by just 2–3%. Furthermore, from our data a higher score on Treatment Control (hypothesized as increasing the chance of poor recovery) showed the opposite. This is not in line with other research in patients attending a general physician, an inpatient rehabilitation program, or an acupuncturist for low back pain, where reporting higher scores for IPs was predictive of greater limitations in PF with low back pain [28–31]. We researched outpatients receiving usual physiotherapy care for a wide range of MSP, which makes comparison of results difficult. Looking at the difference between good and poor clinical recovery for Treatment Control scores (Table 4) we see very small differences. This means that, although Treatment Control contributes to added predictive value, the clinical importance is limited. In contrast with previous research, we adjusted our findings for known generic prognostic factors and psychological factors.

The IP Timeline (patients’ beliefs about how long their condition will last) is an additional prognostic factor of poor recovery in PF (Table 6). This is in line with published research about recovery expectations, in which Timeline was found to be a factor in general expectations for individual recovery [32].

For interpretation of our findings on the additional predictive value of baseline IPs, the chosen generic prognostic factors must be taken into account. Using other prognostic factors may lead to different outcomes and interpretation of the predictive value of baseline IPs.

### Association and difference in predictive value between 4DSQ and brief IPQ-DLV

The weak associations of the Brief IPQ-DLV with the 4DSQ indicate that they address different constructs. Additionally, both performed equally weakly as predictors for poor recovery in all three clinical outcomes. This indicates that the Brief IPQ-DLV (9-items) could not be replaced by the 4DSQ (50-items), and that neither makes a clinical contribution of added predictive value for poor recovery.

### Limitations and strengths

First, despite the large number of participating primary care physiotherapy centers, selection bias may have occurred. Gender differences are reported for increased female risk of chronic pain and more severe pain [33]. This might be of influence on the outcome since 68.9% of our population was female. Additionally, we have no information about patients who were invited but did not participate. Further, we used the Brief IPQ-DLV and, although this is frequently used [8], it is debatable whether dimensions of beliefs about MSP can be measured with questionnaires alone [34]. Qualitative research might add extra in-depth information, but this was outside the scope of this study. Finally, the general prognostic factors were based on a systematic review among a range of musculoskeletal disorders [2]. Though this suited our population well, it is possible that we have overlooked other general relevant factors, such as sleep or central sensitization.

A strength of this study is that it is the first multicenter study done in primary care physiotherapy centers, with 28 primary care physiotherapy centers, geographically spread throughout the Netherlands. Hence, our findings are generalizable to patients in private practice in the Netherlands. Secondly, according to Hayden et al.’s criteria [35], our design is the first Phase 3 outcome prediction study focusing on the added predictive value of IPs. A systematic review of association and prognosis of IPs in MSP reported no other similar studies [9]. Thirdly, although there were missing data, the highest rate was 11%, making our dataset robust enough without the need for imputation. As this is the first paper to report on IPs and poor recovery in primary care physiotherapy, we built exploratory models based on univariate *p*-values (Table 5). To overcome the issue of excluding possible relevant IPs we set the *p*-value threshold to 0.10.

### Practical implications/future directions

Overall, the additional contribution of the two IP dimensions, Treatment Control and Timeline, to predictions of poor recovery after three months of usual physiotherapy care were small, the increase in the AUC being only 2–3%. Based on these results**, assessing baseline IPs, over and above the known generic prognostic factors, does not result in a substantial improvement in the prediction of poor recovery.** In addition, the baseline outcome score of the Brief IPQ-DLV does not indicate the use of the questionnaire as a baseline predictor of poor recovery.

However, this does not rule out a value for IPs in MSP, as their possible role as mediators has yet to be researched. Other research designs, such as Single-Case Experimental Designs, have been shown to be of value when looking for relevant factors for recovery from low back pain [36, 37].

In this study, treatment followed KGNF guidelines or, when not relevant, the physical therapist’s usual practice. Therefore, specific interventions aimed at patients’ beliefs cannot be assumed to have taken place. This could influence existing poor recovery outcomes of 39% for PI, 55% for PF and 30% for GPE. Tailoring interventions that match specific risk factors and patients’ needs has recently brought forward as a preventative strategy for the transition of acute to chronic low back pain [38], so matching interventions with patients’ high baseline IPs is conceivable. We recommend future research into the feasibility and effectiveness of an illness perception-based physiotherapy intervention for patients with disabling MSP.

## Conclusion

Based on the findings of this explorative study, assessing baseline IPs, over and above the known generic prognostic factors, does not result in a substantial improvement in the prediction of poor recovery. Also, no recommendations can be given for preference between the 4DSQ and the Brief IPQ-DLV to assess psychological factors.

The role of IPs as possible mediators has still to be researched. We recommend future research with suitable designs that can look at changeability and possible effectiveness of high IPs on PI, PF and GPE in patients with musculoskeletal pain.

## Data Availability

The datasets used and/or analyzed during the current study are available from the corresponding author on reasonable request.
